# Sickness Presenteeism Predicts Suboptimal Self-Rated Health and Sickness Absence: A Nationally Representative Study of the Swedish Working Population

**DOI:** 10.1371/journal.pone.0044721

**Published:** 2012-09-11

**Authors:** Marina Taloyan, Gunnar Aronsson, Constanze Leineweber, Linda Magnusson Hanson, Kristina Alexanderson, Hugo Westerlund

**Affiliations:** 1 Stress Research Institute, Stockholm University, Stockholm, Sweden; 2 Department of Psychology, Stockholm University, Stockholm, Sweden; 3 Department of Clinical Neuroscience, Karolinska Institutet, Stockholm, Sweden; University of Pennsylvania, United States of America

## Abstract

**Background:**

Earlier studies have suggested that sickness presenteeism (SP) may be a risk factor for future health problems. The purpose of the present study was to test this in a nationally representative prospective study of Swedish workers.

**Methods:**

Prospective cohort with a representative sample of the Swedish working population surveyed in 2008 and 2010. Odds ratios (OR) with 95% confidence intervals (CI) were calculated using logistic regression.

**Results:**

Those who reported more than 7 days of SP had higher risk of suboptimal SRH compared to those who reported no SP (OR = 5.95; 95% CI 4.98–7.12), also after adjustment for confounders (OR = 1.64; 95% CI 1.30–2.06). Those who reported 1–7 days of SP also had an increased risk before and after adjustments. Inclusion of self-rated physical and psychological work capacity did not attenuate the associations, whereas of emotional exhaustion attenuated the ORs to non-significance for both outcomes, indicating that the health consequences associated with SP are largely related to mental health.

**Conclusion:**

The results strengthen earlier findings suggesting that SP can be a risk factor for future suboptimal general health and sickness absence, particularly through mental health problems. This indicates that asking about SP could yield important information for employers, occupational health practitioners and GPs, possibly leading to more timely intervention that could decrease the risk of future sickness absence and more serious health problems, especially in the mental domain. Further studies of the possible causal pathways between SP and future health development are also warranted, especially since going to work is often seen as desirable also for those with poor health.

## Introduction

The basis for sickness absence is reduced work capacity due to illness. From being completely healthy and able to work the individual is affected by any form of sickness leading to loss of work capacity, which can be acute (e.g. stomach flu), episodic (e.g. migraine) or chronic (e.g. onset of diabetes). The individual must then decide how he or she will act in relation to the illness or loss of work capacity. Sickness absence (SA) is not the only option in this situation; as people can sometimes also choose sickness presence, i.e. – going to work despite feeling unhealthy. Sickness absence and sickness presence (SP) may be said to be mutually exclusive courses of action in case of sickness entailing loss of work capacity.

Two perspectives of SP are predominant in the literature and research [1]. The North American perspective is focused on productivity loss at work related to illness [2], while the Scandinavian and European literature defines SP as attending work despite illness which would have motivated sickness absence, i.e. a person goes to work despite the feeling that he/she should have stayed at home because of his/her subjectively poor health condition [3], [4]. This study is based on latter definition.

Ill health appears not unexpected as the strongest determinant of SP [5]. In cross-sectional studies there is a strong correlation between SP and various health problems. For example, a higher proportion of people with back and neck pain as well as extreme fatigue can be found in groups with high SP [6]. Cross-sectional studies have shown associations between both individual and work-related conditions and frequency of SP as income [6], sick pay [7], job insecurity [8], ease of replaceability [5], [9], sick leave policy [10], presenteeism cultures [11], [12], [13], adjustment latitudes [14], downsizing [15], stress and high job demands [16], [17] and low social support [16].

To answer the question if SP is a risk factor for future illness and sick leave longitudinal studies are needed and some such studies have been made in recent years - mostly in Europe. There are at least six such studies.

As regards the risk of future ill health self-rated health (SRH) is often used as the main outcome measures but a number of more specific outcomes that vary between studies have also been used. One Swedish study found that SP at baseline was an independent risk factor for poorer health both at 18 months and at 36 months follow-up, even when a number of potential confounders at baseline were controlled for [18]. The result was the same in the two different materials: female-dominated work in the public sector and male-dominated employment in the private sector, suggesting that the results had relatively high validity. Similar results emerged from another Swedish prospective cohort study of working people between 25 and 50 years [19]. The most obvious negative effect of SP was related to later physical complaints and to work ability, but a dose-response effect was found for a number of other more specific problems. A longitudinal three wave study from Netherland showed that high demands with subsequent SP was a risk behavior for burn-out [19].

As regards the risk that SP may increase the risk for future SA there are a few studies. A Swedish study showed that high SP (more than 5 times) during the previous year was a risk factor for future SA (more than 30 days) after two and three years also after controlling for a number of health-related lifestyle, demographic and work-related variables at baseline [18]. In a large Danish study based on a random sample (n  = 12,000), researchers found that people who had gone to work more than 6 times when they were ill the year before baseline had a 74 percent higher risk of SA longer than 2 months after controlling for confounders and health at baseline [20].

Conclusions on SP and ill health have also been drawn from other types of studies. In a study of the well-known Whitehall cohort [21], two groups of employees with poor health due to heart disease were compared. In one group, the participants had continued to work as usual (the SP group) while people in the second group had a SA period after the disease. In follow-up analysis after three years, with control for known risk factors for cardiovascular disease the researcher found that the risk of heart attacks had doubled in the SP group compared with the second group who had had low or moderate SA. In a prospective study of SP and cardiovascular disease in the same cohort, however, the authors found no evidence for SP acting as trigger [22].

The cited studies have generated hypotheses about the mechanism connecting SP and illness and diseases. Stress and hard physical work during illness could thus possibly trigger a more serious medical event, such as myocardial infarction [21], [23], [24], although the relevance of triggering for non-manual work has been challenged [22]. A second possibility is that lack of necessary rest and recuperation could prolong the course of some diseases, and possibly lead to increased risk of sequelae. Furthermore, working while ill might contribute to cumulative psychological burden with long-term pathophysiological consequences for the development of disease, consistent with allostatic load hypothesis [25]. An additional hypothesis is that SP, instead of being causal agent, is a marker of a lifestyle in which symptoms are ignored and medical care is not sought when needed [26].

Research on presenteeism and SP has recently been reviewed by Johns (2010) [27]and earlier by Schultz & Edington (2007) [2]. The research field has grown rapidly during the last decade and forms a new research area in its own, but it also has high relevance for the understanding and interpretation of SA research. The purpose of his study was to investigate whether SP can predict suboptimal SRH and self-reported SA two years later and to identify which aspects of health are most prone to be influenced by SP.

## Materials and Methods

### SLOSH and Study Population

This prospective study is based on the Swedish Longitudinal Occupational Study of Health (SLOSH), a cohort survey initially representative of the working population in Sweden in 2003–2005, commissioned by the Stress Research Institute at Stockholm University. The surveys were administrated by Statistics Sweden, starting in 2006, with follow-ups in 2008 and 2010. This study is based on the 7,445 participants in the 2008 survey who were working and who also answered the 2010 questionnaire. The participants were fully informed about the study through the invitation letters, and returning of a filled-out questionnaire was interpreted as informed consent. The Regional Research Ethics Board in Stockholm approved this survey.

### Dependent Variables

Two outcomes from 2010 were analyzed in this study: self-rated health and sickness absence. Both of them were based on a single question.

*Self-rated health* was based on the well-validated question on *“How do you perceive your general health?”* with five response alternatives, here dichotomized into: (1) good (good or very good) versus (2) suboptimal (fairly bad, bad, and very bad).

*Sickness absence* was measured by the question *“Approximately how many days in total have you been on sick leave during the past 12 months?”* with five response options, here dichotomized into absence (1) 7 days or less (not at all, or 1–7 days) and (2) more than 7 days (8–30 days, 31–90 days, and 91 days or more). When sickness absence 2008 was taken into account this variable was categorized into three groups: 1) not at all; 2) 1–7 days and 3) more than 7 days a year.

### Independent Variable

*Sickness presenteeism* was based on the question “For roughly *how many days in total during the last 12 months did you go to work despite thinking that you should have reported in sick considering your health status*?” The responses in 2008 were categorized as (1) not at all, (2) less than 8 days (1–7 days) versus (3) more than 7 days a year, consisting of three response options (8–30 days, 31–90 days and 91 days and more).

### Covariates

As *socio-demographic characteristics* in 2008, the following variables derived from 2008 were included in some analyses: *Age* and *Education* as continuous variables (in years), *income* divided into three proportionally similar tertiles. *Education* was originally coded as 1) primary school (≤9 years); 2) intermediate education (10–12 years) and 3) higher education (>12 years), but was transformed into a continuous variable (years of education) before inclusion in the statistical analyses.

As *work related factors*: we used *Satisfaction with work tasks* (“*How satisfied or dissatisfied are you with your current work tasks?*”) from 2008 which was categorized as 1) satisfied (very satisfied, fairly satisfied) and 2) dissatisfied (fairly dissatisfied, very dissatisfied).

Three *lifestyle-related factors* measured in 2008 were included in this study: Body mass index (BMI), current smoker (*“Do you smoke?”* with answers “yes” (Yes, daily; Yes, occasionally) and “no” (“No”)) and physical activity (*“How much exercise do you get? Include any walking or cycling you do to work”* categorized: “never/very little” (“Never exercise”; “Very little exercise, take some walks”) and “occasionally/regularly” (exercise occasionally”, “exercise regularly”). BMI was calculated as weight in kilograms divided by height in metres squared and categorized into three groups (normal, overweight and obese) according to guidelines for adults [28]. In addition, satisfaction with life based on the question *“All things considered how satisfied or dissatisfied are you with your life in as a whole?”* was included into the study. Those who reported themselves as “very dissatisfied”, “relatively dissatisfied”, “slightly dissatisfied” and “neither satisfied nor dissatisfied” were categorized as dissatisfied with life.

*Health factors* included from 2008 and/or 2010 were physical work capacity, psychological work capacity, and sleep quality categorized as 1) good (very good, fairly good) and 2) suboptimal (neither good nor bad, fairly bad, and very bad). *Musculoskeletal pain* comprised three questions referring to time after work and three different locations: 1. Pain in the upper part of spine or neck; 2. Pain in the lower back; 3. Pain in shoulders or arms. Each of these items had five response alternatives: “every day”, “a couple of days a week (1 day of 2), “once a week (1 day of 5)”,”a couple of days a month (1 day of 10)”, “not at all/seldom during preceding 3 months”. All values were added to form an overall pain index, which was based on cut-offs and coded as 1) often; 2) sometimes and 3) seldom.

*Additionally, emotional exhaustion* in 2010- one of the three dimensions of burnout (exhaustion, cynicism and inefficacy) [29], was based on subscale from the Burnout Inventory Scale developed by Maslach and considered to be the core symptom of the burnout syndrome [30]. Emotional exhaustion was measured by five items referring to emptiness, tiredness and feelings of to have job burnout, with six response alternatives (from “every day” to “a few times a year or less/never”). A sum of all items was calculated and included into the analyses as a continuous variable.

### Statistical Analyses

Bivariate and cross lagged associations among SRH, SP and SA 2008 and 2010 were assessed by Spearman correlations, shown in Table 1. Calculations of the prevalence of the outcome variables (SRH and SA) were performed separately for three groups of SP. Pearson’s chi2-tests were used to test the level of statistical significance of differences in prevalence of outcome in variables including into this study (Table 2 and Table 3). Unconditional logistic regression was used to calculate the odds ratios (ORs) and 95% confidence interval (95% CI) in four models as shown in Table 4 and Table 5. In the first model, age, sex and income were included in addition to SP. In order to identify the strongest confounder one baseline covariate was added at a time in the same model (all shown in Table 4 and Table 5). The fully adjusted model (M2) is presented after adjustment for all covariates simultaneously in Table 4 and Table S1. Only significant variables were introduced into the fully adjusted model. Table 5 introduced specific health measures from 2010 (M3a-c) one at a time in addition to previously included covariates from 2008. This was not done as an additional adjustment for confounders but in order to assess which aspects of the more general measures of health used as outcomes were most influenced by previous SP. In the full model (M 4) for both outcomes (SRH and SA) all covariates were included together. The fit of the models was judged by the Hosmer-Lemeshow goodness-of-fit test. The models were considered acceptable if p>0.05, and all models met this demand [31]. All analyses were performed with Stata version 11 (College Station, TX, USA).

**Table 1:** Spearman correlations between the major independent and dependent variables in the study.

	1	2	3	4	5	6
1.Sickness Presenteeism 2008	1.00					
2.Self-rated health 2008	0.32*	1.00				
3.Sickness Absence 2008	0.25	0.21	1.00			
4.Sickness Presenteeism 2010	0.50*	0.27	0.18	1.00		
5.Self-rated health 2010	0.26	0.58*	0.18	0.31*	1.00	
6.Sickness Absence 2010	0.18	0.18	0.45*	0.24	0.21	1.00

* Statistically significant.

**Table 2:** The distribution (% and mean) of demographic characteristics in total and among individuals reporting Sickness Presenteeism 2008, Suboptimal Self-rated Health 2010 and Sickness Absence (>7 days) 2010 respectively with test of statistical differences (*P* value), n = 7,445.

	Total,column %	Sickness Presenteeism2008			P value	SuboptimalSRH, 2010,	P value	Sickness Absence, 2010, >7 days	P value
Variable in 2008		Not,at all	1–7 days	>7 days					
Total sample		35.7	51.3	13.0		20.5		18.7	
**Age,** mean (SD) (years)	49.4 (10.32)				<0.0001		0.043		0.224
≤30	4.4	24.5	60.4	15.1		19.9		21.7	
31–40	17.0	27.2	58.0	14.8		19.0		18.6	
41–50	28.3	32.1	53.1	14.8		21.3		18.0	
51–60	34.1	38.1	49.8	12.2		21.8		19.7	
61–70	16.2	49.3	41.9	8.8		18.0		16.5	
**Sex**					0.001		<0.05		<0.0001
Women	56.1	34.6	51.2	14.2		19.3		22.4	
Men	43.9	37.1	51.5	11.4		22.0		14.1	
**Education** (years)					<0.0001		<0.0001		<0.05
Primary (<10)	9.8	43.3	43.6	13.1		22.0		18.9	
Intermediate (10–12)	51.8	35.6	50.0	14.4		22.5		20.0	
High (>12)	38.4	34.0	55.0	11.0		17.4		16.9	
**Income** (x1000 SEK) mean (SD)	309.81 (149.18)				<0.0001		<0.0001		<0.0001
0–245	33.3	34.1	49.5	14.4		22.7		24.0	
246–323	32.9	34.4	52.3	13.3		20.9		19.5	
>323	33.8	38.6	52.2	9.2		17.9		12.1	
**Sickness Presenteeism**							<0.0001		<0.0001
Not,at all	35.7					11.9		13.2	
1–7 days	51.3					20.2		18.1	
8–30 days	11.9					39.4		34.0	
31–90 days	1.1					67.9		52.2	
≥91 days	0.9					70.8		41.8	
**Sickness Absence**					<0.0001		<0.0001		<0.0001
Not, at all	44.9	46.2	47.3	6.5		15.8		7.1	
1–7 days	36.2	29.5	57.5	13.0		19.4		18.7	
8–30 days	13.8	19.8	54.5	25.7		30.0		46.7	
31–90 days	3.1	26.5	38.3	35.2		37.4		48.2	
≥91 days	2.0	37.8	25.8	36.4		44.8		50.5	

**Table 3:** The distribution (%) of all covariates in total and among individuals reporting Sickness Presenteeism, Suboptimal Self-rated Health and Sickness Absence (>7 days) respectively with test of statistical differences (P value), n = 7,445.

	Total,column %	Sickness Presenteeism 2008			P value	Suboptimal SRH,2010, row	P value	Sickness Absence, 2010, row	P value
Variable		Not,at all	1–7 days	>7 days					
**Physical work capacity 2008**					<0.0001		<0.0001		<0.0001
Good	80.4	38.1	51.4	10.5		14.7		16.8	
Suboptimal	19.6	26.0	50.8	23.2		43.8		26.5	
**Satisfaction with work tasks 2008**							<0.0001		<0.0001
Yes	90.9	37.0	50.9	12.1	<0.0001	19.4		18.0	
No	9.1	23.4	53.9	22.7		31.5		25.3	
**Psychological work capacity 2008**							<0.0001		<0.0001
Good	84.7	37.8	51.6	10.6	<0.0001	16.6		17.3	
Suboptimal	15.3	24.3	49.6	26.1		41.1		26.6	
**Musculoskeletal pain 2008**					<0.0001		<0.0001		<0.0001
Low	41.4	22.0	54.1	23.9		11.7		13.2	
Moderate	27.3	33.4	56.2	10.4		18.3		18.2	
Severe	31.3	47.2	46.8	6.0		32.8		26.3	
**Sleep quality 2008**					<0.0001		<0.0001		<0.0001
Good	66.7	41.6	50.3	8.1		13.4		16.3	
Suboptimal	33.3	24.0	53.3	22.7		34.5		23.5	
**Life satisfaction 2008**					<0.0001		<0.0001		<0.0001
High	87.7	37.6	51.6	10.8		16.9		17.8	
Fair	9.4	23.8	51.2	25.0		42.2		22.6	
Low	2.9	20.6	44.0	35.4		51.9		35.0	
**SRH 2008**					<0.0001		<0.0001		<0.0001
Good	79.5	40.0	51.7	8.4		11.7		15.8	
Suboptimal	20.5	16.7	49.8	33.5		59.6		32.3	
**Current smoker 2008**					<0.0001		<0.0001		<0.05
Yes	14.6	36.5	51.4	12.1		25.2		22.0	
No	85.4	32.0	50.7	17.3		19.6		18.1	
**Physical activity 2008**							<0.0001		<0.05
Occasionally/regularly	82.7	37.2	50.7	12.1		17.8		18.2	
Never/very little	17.3	29.0	54.1	17.0		33.6		21.0	
**Body mass index (BMI) 2008**					<0.0001		<0.0001		<0.0001
Normal (<25)	49.6	37.4	51.8	10.8		15.9		16.7	
Overweight (25–29.9)	38.5	34.5	51.8	13.7		22.4		17.9	
Obesity (≥30)	11.9	32.3	48.4	19.3		32.0		28.5	
**Physical work capacity 2010**							<0.0001		<0.0001
Good	64.2					8.3		13.4	
Suboptimal	35.8					27.3		21.8	
**Psychological work capacity 2010**							<0.0001		<0.0001
Good	41.4					11.1		14.0	
Suboptimal	58.6					27.0		22.0	
**Emotional Exhaustion 2010**							<0.0001		<0.0001
Low	26.4					7.3		11.5	
High	73.6					25.0		21.0	

**Table 4:** Odds ratios (OR) with 95% Confidence Intervals (95% CI) of suboptimal Self-rated Health and Sickness Absence in two groups of Sickness Presenteeism with changes of OR and separate inclusion of one variable at a time and final model (M2) adjusted for all covariates together, n = 7445.

	Self-rated Health 2010			Sickness Absence 2010	
Predictors 2008	M1–M0 (crude) +age+sex+education +income,OR,95% CI	M2 Fully adjusted M1+ all covariates listed below*		M1–M0 (crude) age+sex+education +income,OR,95% CI	M2 Fully adjusted M1+ all covariates listed below*
**Sickness presenteeism**					
Not, at all	Reference	Reference		Reference	Reference
1–7 days	1.96*** (1.69–2.26)	1.26** (1.06–1.49)		1.44***(1.23–1.68)	1.10 (0.91–1.29)
>7 days	5.95*** (4.98–7.12)	1.64*** (1.30–2.06)		3.57*** (2.95–4.33)	1.46** (1.15–1.86)
**Change in OR (%) for the different levels of the exposure when covariates are added separately one by one (in preparation for including in M2)**					
**Predictors 2008**	**SP 1–7 days**	**SP>7 days**	**SP 1–7 days**		**SP>7 days**
Physical work capacity	−17	−24	±0		−12
Psychological work capacity	−9	−19	±0		±0
Sleep quality	−27	−35	−13		−14
Musculoskeletal pain	−28	−35	−23		−22
Satisfaction with work tasks			−11		±0
Life satisfaction	−11	−38	±0		±0
SRH 2008	−45	−65	−23		−32
Sickness absence 2008	±0	−17	−52		−59
**In total**	−73	−87	−77		−82

* Smoking, physical activity and BMI did not impact OR of SP and therefore are not shown in the table;

** P<0.05,

*** P<0.001.

**Table 5:** The Result of Logistic Regression with inclusion of Aspects of the Outcomes (M3a –additional adjustment for Self-rated Physical Work Capacity; M3b- for Self-rated Mental Work Capacity and M3c – for Emotional Exhaustion: M4– fully adjusted model).

	Suboptimal Self-rated Health (SRH) 2010			Sickness Absence (SA) 2010		
	M3a-c – M2+ one additional covariate from 2010 at a time		M4 fully adjusted M3a-c+ all covariates listed below	M3a-c – M2+ one additional covariate from 2010 at a time		M4 fully adjusted M3a-c+ all covariates listed below
	OR, 95% CI	Δ OR, %	OR, 95% CI	OR, 95% CI	Δ OR, %	OR, 95% CI
**Sickness Presenteeism 2008**		Days 1–7/>7			Days 1–7/>7	
Not at all	Reference		Reference	Reference		Reference
1–7 days	1.26* (1.06–1.49)		1.15 (0.94–1.42)	1.10 (0.91–1.29)		1.02 (0.85–1.22)
>7 days	1.64** (1.30–2.06)		1.28 (0.97–1.69)	1.46 *(1.15–1.86)		1.26 (0.98–1.62)
**Physical work capacity 2010**						
Good	Reference			Reference		
Suboptimal	3.65** (3.10–4.32)	+12/+12		1.31*(1.10–1.60)	±0/±0	
**Psychological work capacity 2010**						
Good	Reference			Reference		
Suboptimal	3.27** (2.70–3.97)	+15/+14		1.40*(1.12–1.75)	±0/±0	
**Emotional exhausion 2010**						
**High**	Reference			Reference		
**Low**	0.89**(0.89–0.91)	−58/−66		0.96** (0.95–0.98)	±0/−98	
**In total**		−42/−56			±0/−52	

* P<0.05,

** P<0.001.

## Results

The correlations between self-reported health, sickness presenteeism and sickness absence are shown in Table 1. The distribution of all independent and adjustment variables is shown in Table 2. There was a slightly higher proportion of women (56.1%), the mean age was 49 years (SD 10.3). In 2008, more than one third (35.7%) of the participants reported no SP at all during the past 12 month, while about half (51.3%) reported 1–7 days of SP. About 66% of the sample reported suboptimal sleep quality and one third of them had often had musculoskeletal pain after work. Further, in 2008, 18.2% reported suboptimal general health and in total 55% had been sickness absent (36.2% in 1 to 7 days and 18.8% >7 days). In 2010, the prevalence of self-reported suboptimal health was 20.5% and 18.7% had been absent from work more than 7 days during the last 12 months. Odds ratio of suboptimal self-rated health in 2010 was substantially increased for those who had reported more than 7 days of SP in 2008 compared to those reporting no SP (OR = 5.96; 95% CI 4.98 to 7.12) after adjustment for age, sex, and income, and OR = 1.46 (95% CI 1.15 to 1.86) in the fully adjusted model (Table 4, left). SP of 1–7 days was associated with an increased risk of suboptimal self-rated health with OR = 1.96 (95% CI 1.69 to 2.26) after adjustment for age, sex, and income and OR = 1.26 (95% CI 1.06–1.49) after adjustment for all covariates. Adjustment for self-rated health in 2008 attenuated the odds of suboptimal self-rated health in the high SP group to the largest extent with 65% (OR = 4.79; 95% CI 4.06 to 5.67) followed by sleep quality, musculoskeletal pain, life satisfaction, and physical as well as psychological work capacity. On the other hand covariates as age, education, income and smoking became non-significant after adjustments in the final model.

Another pattern was observed regarding SA. The risk of more than 7 days of SA during the last 12 months reported in 2010 was substantially increased for those who had reported 7 days or more of SP in 2008 (OR = 3.57 95% CI 2.95 to 4.33) after adjustment for age, sex, and income, and OR = 1.46 (95% CI 1.15–1.86) in the fully adjusted model (Table 4, right). However, the excess risk for those who had reported less than 7 days of SP (OR = 1.44, 95% CI 1.23 to 1.68) disappeared after full adjustment for the included confounders. The confounders which most attenuated the risk estimates for SA in 2010 were SA in 2008 followed by SRH in 2008.

Table 5 reports how adjustment for different health indicators from 2010 influenced the risk estimates for SRH and SA. Inclusion of self-rated physical and psychological work capacity did not attenuate the associations, whereas emotional exhaustion attenuated the ORs to non-significance for both outcomes.

## Discussion

The main results of this study were that sickness presenteeism (SP), i.e. attending work despite illness that the respondent believes (s)he should have stayed home for, predicts suboptimal SRH and sickness absence (SA) two years later, even after adjustment for multiple possible confounders at baseline. These associations seem largely to be mediated by an increased risk of emotional exhaustion at follow-up (2010) among those who had attended work despite illness. In addition, sex, low income, musculoskeletal pain, poor sleep quality, self-rated physical work capacity, suboptimal SRH, life dissatisfaction and overweight/obesity measured at baseline were significantly and independently related to suboptimal SRH and SA after two years. In accordance with previous studies in Sweden, SP predicted both future poor self-rated health [18] and SA [32], [33].

Our results are in agreement with a study which showed that physical health complaints and/or diseases have an impact on general SRH and SA. Associations between ill health and SA, and between socioeconomic status and SA, were found in the Whitehall II study which includes both men and women from 20 civil service departments in London. Furthermore, baseline health strongly predicted rates of long–term sickness absence [34]. Aronsson found that among those with high SP there was significantly higher number of persons with upper back/neck pain, fatigue, slightly depressed mood, and the levels of SA was higher among those with high SP [6]. Although we investigated the relationship between SP and ill health and SA, we found almost the same pattern in our study. Inclusion of poor health at baseline (in 2008) attenuated the odds for reporting poor health two years later with as much as 45% and 65% among those who had reported high SP. Further, SA at baseline explained 52% and 59%, respectively, of the excess risk for future SA.

Approximately 64% of the participants in the current study reported SP over a 12-month period, 51% of them for 1–7 days. It has been shown previously that educational level is another factor associated with SP [35]. Contrary to this register study, in our study the prevalence of reporting SP was higher among individuals with intermediate education than the proportion of SP in low and high educated respondents. On the other hand, education was a significant confounder when age, sex, and income were taken into account in the logistic regression model: highly educated subjects had 7% lower ORs for reporting SP than those with lower education. This significance disappeared when other confounders were including into further models. However, the total of the 64% of SP reported in our study is similar to the results in a Danish sample of 12,835 employees which showed that more than 70% reported SP at least once during a 12-month period. The same study indicated that work-related factors were slightly more predictive of SP than conservative attitudes to absence or personal circumstances [20]. We lacked assessments of personal circumstances and attitudes in the current study; however, we included several health-related factors. Some studies have previously shown that SP is correlated with sickness absence and illness [5], [9], [36]. Gustafsson and Marklund suggest that both SP and SA are strong predictors of future poor health and that SP appeared to lead to SA, whereas SA did not have any effect on future SP [19]. According to a recent a study based on the SLOSH cohort, there was a strong cross-sectional association between SA and SP even after a large number of adjustments for indicators of health and work environment, indicating that SP may be more than a behavioral alternative to SA [37]. Possible explanations for this association include an individual tendency to regard absence as an appropriate response also to minor diseases, which could be an alternative explanation for the prospective association found in the present study. We considered that SP might additionally predict SRH and SA in employed respondents. When individuals feel too ill to work, they can sometimes choose either SA or SP. In the present study we observed that SP predicts SA and suboptimal SRH two years later, also after adjustment for a large number of factors, which may suggest causality. This is in agreement with two previously published Swedish follow-up studies [18], [32]. A number of studies, however, have concluded that SA may not be the best alternative for individual health: while in some cases it may be restorative, in other it can lead to social isolation and poor personal finances [38], [39].

### Strengths and Limitations

The large study population and the prospective design are the main strengths of this study. A further strength is the more comprehensive adjustment for possible confounders than in previous studies of the possible health consequences of SP, including work-, health-, and lifestyle-related factors.

The novelty of our study is the investigation of SP as a predictor of suboptimal SRH in nationally representative sample of Swedish working population. Another strength of the present study compared to other studies is using of a holistic perspective to explore this issue by including relevant health- and lifestyle-related factors besides work-related factors.

The main limitation of this study is that all variables are self-reported. This could cause bias due to spurious correlations between SP and the studied outcomes. It has been suggested that subjective health complaints (illness) are fairly distinct from medically observable disease, which in turn is different from the social consequences of poor health (sickness) (46). However, SRH has repeatedly been shown to be one of the best predictors of mortality [40], [41], indicating that it does capture important aspects of objective health. Despite this, it is possible that the prospective association between SP and future health is driven by unmeasured aspects of health rather than by a causal effect of SP.

As this is a cohort study, not a randomized controlled trial, it is not possible from this type of study to know how the SHR or SA would have been if the individuals with SP in 2008 had taken SA instead. Nor is it possible to draw conclusions about the future SHR or SA among those SA in 2008– if they had chosen to be SP instead. To gain more knowledge about these aspects, different types of studies are warranted, e.g. more prospective cohort studies, with repeated measurements, case-crossover studies, and intervention studies.

However, subjective health has been shown to be important predictor of objective morbidity even if it has been concluded that illness, sickness and disease represent different aspects of morbidity [42].

### Conclusions

The results strengthen earlier findings suggesting that SP can be a risk factor for future suboptimal general health and sickness absence, particularly through mental health problems. This indicates that asking about SP could yield important information for employers, occupational health practitioners and GPs, possibly leading to more timely intervention that could decrease the risk of future sickness absence and more serious health problems, especially in the mental domain. Further studies of the possible causal pathways between SP and future health development are also warranted, especially since going to work is often seen as desirable also for those with poor health.

## Supporting Information

Table S1 (PDF)
