# Supplementary material for: Sickness Presenteeism Predicts Suboptimal Self-Rated Health and Sickness Absence: A Nationally Representative Study of the Swedish Working Population
Source: PLoS One. 2012 Sep 11;7(9):e44721. doi: 10.1371/journal.pone.0044721 (PMC3439368; doi:10.1371/journal.pone.0044721)
Supplement: Table S1 — (PDF) [file pone.0044721.s001.pdf]

Webbtable 4a. Odds ratios (OR) with 95% Confidence Intervals (95% CI) of suboptimal Self-rated Health and Sickness Absence in three groups of Sickness Presenteeism with changes of OR and separate inclusion of one confounder at time and final model (M3) adjusted for all control variables together, n=7445

Predictors 2008	Suboptimal Self-rated Health 2010				Sickness Absence 2010			
	M1- M0 + age + sex + education + income	M2a-j- M1 + one additional confounder at a time		M3 fully adjusted	M1- M0 + age + sex + emp + income	M2a-x - M1 + one additional confounder at a time		M3 fully adjusted
	OR, 95 % CI	OR, 95 % CI	Δ OR, %	OR, 95 % CI	OR, 95 % CI	OR, 95 % CI	Δ OR, %	OR, 95 % CI
**Sickness presenteeism**			Days 1-7/>7				Days 1-7/>7	
Not at all	Reference			Reference	Reference			Reference
1-7 days	1.96** (1.69-2.26)			1.26* (1.06 -1.49)	1.44** (1.23-1.68)			1.10 (0.91-1.29)
> 7 days	5.95 ** (4.98-7.12)			1.64** (1.30-2.06)	3.57** (2.95-4.33)			1.46 * (1.15-1.86)
**Age** (continuous)	1.00* (1.00-1.01)			0.99 (0.98-1.00)	1.00 (0.99-1.01)			1.00 (0.99-1.01)
**Sex**								
Women	Reference			Reference	Reference			Reference
Men	1.31** (1.15-1.49)			1.34** (1.14-1.59)	0.73** (0.63-0.84)			0.79* (0.67-0.94)
**Education** (continuous)	0.93* (0.88-0.98)			1.00 (0.94-1.08)				
**Income** (x1000 SEK)								
0-245	Reference			Reference	Reference			Reference
246-323	0.87 (0.76-1.03)			0.90 (0.76-1.08)	0.74** (0.63-0.86)			0.74 * (0.62-0.89)
>323	0.77* (0.65-0.91)			0.86 (0.71-1.05)	0.47** (0.39-0.57)			0.58** (0.48-0.71)
**Physical work capacity**								
Good		Reference	-17/-24	Reference		Reference	±0/-12	Reference
Suboptimal		4.00 (3.50-4.53)		2.02** (1.70-2.39)		1.48 (1.27-1.73)		1.04 (0.86-1.27)
**Psychological work capacity**								
Good		Reference	-9 /-19	Reference		Reference	±0/±0	Reference
Suboptimal		2.87 (2.48-3.31)		1.17 (0.97-1.40)		1.34 (1.13-1.59)		0.98 (0.79-1.21)
**Sleeping quality**								
Good		Reference		Reference		Reference	-13/-14	Reference
Suboptimal		2.87 (2.54-3.25)	-27/-35	1.71** (1.47-1.99)		1.31 (1.14-1.50)		1.05 (0.89-1.24)
**Musculoskeletal pain**								
Low		Reference	-28/-35	Reference		Reference	-23/-22	Reference
Moderate		1.53 (1.29-1.81)		1.33 * (1.11-1.61)		1.31 (1.11-1.56)		1.14 (0.94-1.38)
Severe		2.87 (2.45-3.35)		1.78 ** (1.48-2.13)		1.69 (1.43-1.99)		1.36* (1.13-1.63)
**Satisfaction with work tasks**								
Satisfied						Reference	-11/±0	Reference
Dissatisfied						1.29 (1.05-1.60)		1.16 (0.91-1.48)
**Life satisfaction**								
High		Reference	-11/-38	Reference		Reference	±0/±0	Reference
Fair		2.95 (2.48-3.50)		1.66** (1.34-2.06)		1.11 (0.90-1.38)		0.91 (0.70-1.17)
Low		4.06 (3.02-5.45)		1.61* (1.12-2.30)		1.95 (1.39-2.73)		1.41 (0.94-2.11)

**SRH**						
Good	Reference	-45/-65	Reference	Reference	-23/-32	Reference
Suboptimal	9.02 (7.82-10.39)		4.79** (4.06-5.67)	1.98 (1.69-2.32)		1.41* (1.15-1.74)
**Sickness absence**						
No	Reference	±0 /-17	Reference			
Yes	1.76 (1.53-2.03)		1.26* (1.06-1.49)			
**Sickness absence**						
Not,at all				Reference	-52/-59	Reference
1-7 days				2.57 (2.15-3.08)		2.58** (2.13-3.12)
8-30 days				8.75 (7.14-10.72)		8.21** (6.61-10.18)
31-90 days				8.74 (6.30-12.13)		7.92** (5.58-11.25)
91 days or more				9.82 (6.48-14.89)		8.54** (5.42-13.45)
**Current smoker**						
No	Reference	±0 /±0	Reference			
Yes	1.23 (1.05-1.45)		1.11 (0.91-1.34)			
**Physical activity**						
Occasionally/regularly	Reference	±0 /±0	Reference	Reference	±0/±0	Reference
Never/very little	2.15 (1.86-2.48)		1.32* (1.11-1.58)	1.21 (1.02-1.44)		1.03 (0.85-1.26)
**Body mass index (BMI)**						
Normal	Reference	±0 /±0	Reference	Reference	±0/±0	Reference
Overweight	1.39 (1.21-1.59)		1.21* (1.02-1.41)	1.17 (1.01-1.35)		1.05 (0.89-1.24)
Obesity	2.13 (1.78-2.55)		1.56** (1.25-1.93)	1.89 (1.56-2.29)		1.49** (1.19-1.85)
**In total**		-73/-87			-77/-82	
